# Model-Based Meta-Analysis in Psoriasis: A Quantitative Comparison of Biologics and Small Targeted Molecules

**DOI:** 10.3389/fphar.2021.586827

**Published:** 2021-07-01

**Authors:** Huan He, Wenwen Wu, Yi Zhang, Meng Zhang, Ning Sun, Libo Zhao, Xiaoling Wang

**Affiliations:** ^1^ Clinical Research Center, Beijing Children’s Hospital, Capital Medical University, National Center for Children’s Health, Beijing, China; ^2^ Department of Pharmacy, Children’s Hospital of Nanjing Medical University, Nanjing, China

**Keywords:** model-based meta-analysis, moderate to severe psoriasis, biologics, small targeted molecules, efficacy comparation

## Abstract

**Background:** The response time-course information of biologics and small targeted molecules for the treatment of moderate to severe plaque psoriasis which helps clinicians to understand the onset of action and maintenance of effect are unclear. Quantitative information about the efficacy comparation of different systemic agents are needed.

**Methods:** Model-based meta-analysis was conducted and longitudinal models were developed by applying two clinical end points commonly reported in the clinical trials of psoriasis: the proportion of patients achieving ≥75% reduction from baseline Psoriasis Area and Severity Index score (PASI75) and the proportion of patients achieving ≥90% reduction from baseline Psoriasis Area and Severity Index score (PASI90).

**Results:** A total of 80 trials of thirteen biological agents and four small targeted molecules covering 235 treatment arms and 40323 patients with moderate to severe plaque psoriasis were included in this analysis. The drugs were divided into five classes of biologics and three classes of small molecules. Two longitudinal models of PASI75 and PASI90 were used to describe the time-varying drug effect and dose-effect relationship. The typical response-time courses for PASI75 and PASI90 increased over time and finally reached to the platform. For PASI75 end point at week 12, of all the therapeutic drugs, risankizumab administered as 150 mg at week 0, week 4, and q12w showed the most efficacious with PASI75 was 85.95% (95%CI, 75.71–92.60%), followed by ixekizumab administered as 160 mg at week 0, and q4w with PASI75 was 85.9% (95%CI, 76.12–92.79%). As for PASI90 end point at week 12, ixekizumab 160 mg at week 0, and q4w showed the greatest percentage of person achieved PASI90 (67.2%; 95%CI, 49.91–77.2%), followed by risankizumab 150 mg at week 0, week 4, and q12w (65.5%; 95%CI, 47.8–75.7%). What’s more, the risankizumab provided the highest response of PASI90 at week 16 and week 24.

**Conclusions:** This study provided a quantitative efficacy comparation of 17 systemic agents for psoriasis in term of efficacy only and that safety was not considered. Risankizumab and ixekizumab showed superiority for both the two end points.

## Introduction

Psoriasis is a common, chronic, immune-mediated inflammatory skin disease which has a high prevalence worldwide (Boehncke and Schon, 2015; Harden et al., 2015). The prevalence of psoriasis in adults varied from 0.91 to 8.5% between countries (Parisi et al., 2013; Rungapiromnan et al., 2020). It is characterized by chronic inflammatory skin lesions with pruritic, well demarcated, erythematous and scaly plaques combined with a substantial disease burden which affects quality of life (Goff et al., 2015; Cai et al., 2017; Ohtsuki et al., 2019). Treatment options for psoriasis include topical agents, phototherapy and systemic medications such as small targeted molecules and biologics (Menter et al., 2011). The invention of biological agents has greatly improved the treatment outcomes of psoriasis, including inhibitors of tumor necrosis factor (TNF)-α, interleukin (IL)-17, and IL-12/23 (Papp et al., 2008; Saurat et al., 2008; Barker et al., 2011; Paul et al., 2015; Gordon et al., 2016).

There has been rapid development of novel biologic agents for the treatment of moderate-to-severe plaque psoriasis over the past 2 years. IL-23 which is a heterodimer composed of a unique p19 subunit and an associated p40 subunit shared with IL-12 plays a crucial role in the pathogenesis of psoriasis (Chen et al., 2017; Girolomoni et al., 2017). Ustekinumab, one of the IL-12/23 inhibitors which targets the p40 subunit common to both IL-12 and -23 has proved effective in the treatment of psoriasis (Tsai et al., 2011). Recent two years, a new class of biologics for moderate to severe plaque psoriasis in adults which targeted the p19 subunit specific to IL-23 were approved by Food and Drug Administration (FDA) (McKeage and Duggan, 2019). The monoclonal antibody IL-23 inhibitors include guselkumab, tildrakizumab and risankizumab (Gordon et al., 2018; Reich et al., 2017; Reich et al., 2017). The TNF-α and IL-17 inhibitors for the treatment of moderate to severe psoriasis included adalimumab, infliximab, etanercept, certolizumab, secukinumab, ixekizumab, and brodalumab. However, it can be challenging for clinicians to determine how the systemic medications compare with one another.

Several meta-analyses (Armstrong et al., 2020; Erichsen et al., 2020; Warren et al., 2020) were conducted to indirectly compare the relative efficacies of approved biologic agents. However, there are some limitations of the published studies. One of the major limitations is that the efficacy of biologics was assessed at one time point (pooling week 12 or 16 together) in different studies which is the disadvantage of traditional meta-analysis and network meta-analysis method. It can’t provide the time-course response information which helps clinicians to understand the full response profile for different compounds and/or placebo which includes the onset of action and maintenance of effect. Second, the existing meta-analysis covered not all the current systemic agents as eleven biologics including four TNF-α inhibitors, one IL-12/23 inhibitor, three IL-23 inhibitors and three IL-17 inhibitors have been approved for the treatment of moderate-to-severe psoriasis, and some small targeted molecules such as Janus kinase (JAK) inhibitors are investigated.

In order to overcome the problems mentioned above, a novel model-based meta-analysis in psoriasis was conducted. Longitudinal model-based meta-analysis is a meta-analysis that explicitly incorporates the effect of dose and duration using standard pharmacology models and assumptions (Mandema et al., 2011). By encompassing longitudinal data from the literature, it allows the comparation of the effect-time course among different treatment and could provide accurate assessment of the drug response (Checchio et al., 2017). Therefore, it could offer more quantitative information about the data than the traditional and network meta-analyses.

The objectives of our study were to assess the relative efficacy of three IL-23 inhibitors for treatment of psoriasis and provide quantitative information about the comparation of different systemic agents including those approved, discontinued and those under investigation by using longitudinal model-based meta-analysis. Two longitudinal models were developed by applying two clinical end points commonly reported in the clinical trials of psoriasis: the proportion of patients achieving ≥75% reduction from baseline Psoriasis Area and Severity Index score (PASI75) and the proportion of patients achieving ≥90% reduction from baseline Psoriasis Area and Severity Index score (PASI90).

## Methods

### Database Development

A comprehensive search of clinical trials was conducted using PubMed, Cochrane, Embase, and ClinicalTrials.gov websites. The search keywords were as follows: moderate to severe plaque psoriasis, adalimumab, infliximab, etanercept, certolizumab, ustekinumab, briakinumab, guselkumab, tildrakizumab, risankizumab, secukinumab, ixekizumab, brodalumab, apremilast, tofacitinib, baricitinib, alefacept, methotrexate and randomized controlled trial. The cutoff date for the search was July 18, 2019. The articles about psoriasis from the reference lists of prior reviews were also screened.

Inclusion criteria of studies were as following: (i) randomized placebo- or active-controlled trials published in English; (ii) trials including patients which was diagnosed with moderate to severe plaque psoriasis and treated with biologics or small targeted molecules; (iii) studies reported the end point PASI75 (≥75% reduction from baseline Psoriasis Area and Severity Index score) or PASI90 (≥90% reduction from baseline Psoriasis Area and Severity Index score).

For each eligible study, relevant data were extracted including drug name, groups, dose, number of patients, time, efficacy outcomes and subject characteristics. Only the first period data of a crossover trial was included in our analysis. The data of PASI75 and PASI90 of all the published doses were extracted from articles as well as from tables. If efficacy results were presented as a graph, the data were captured by using the GetData Graph Digitizer (V2.25). Two authors (Huan He and Wenwen Wu) independently reviewed the studies and extracted the end point data. Disagreements were settled by a third author (Yi Zhang). In addition, normalization of different dose regimens was conducted to pool the same drug efficacy data.

### Model Development

After graphical exploration of the data, the longitudinal model of PASI75 and PASI90 were developed to describe the dose-effect and time-effect relationships for each drug. All the doses were used for the modelization. The methodology of modeling was reported previously (Checchio et al., 2017; Wu et al., 2019). The structure of longitudinal model was shown below:

$$N_{\text{response},\;ijt}∼\text{binomial }\left(N_{ij},\;P{\left(\text{response}\right)}_{ijt}\right)$$ (1)

$$P{\left(\text{response}\right)}_{ijt}=g\left(E_{0}+E_{\text{drug}}\right)$$ (2)

$$g=\frac{1}{1+\;e^{-\left(E_{0}+E_{\text{drug}}\right)}}$$ (3)

Where response represents the end points PASI75 (≥75% reduction from baseline Psoriasis Area and Severity Index score) or PASI90 (≥90% reduction from baseline Psoriasis Area and Severity Index score). The $N_{\text{response},\;ijt}$ represents the number of patients achieving PASI75 or PASI90 at *t*th time in treatment arm *j* of trial *i*. It follows a binomial distribution with probability P(response) and sample size $N_{ij}$.

The *g* is the inverse logit function to restrict the treatment effect, which is the sum of placebo effect (*E*_0_) and the drug effect (*E*_drug_) to probability scale of a range of 0–1.

The placebo effect (*E*_0_) was described by the parameter intercept (*BSL*), asymptote (*A*) and rate constant of placebo (*k*_pbo_) (Eq. 4).

$$E_{0}=BSL+\;A\cdot \left(1-e^{-k_{\text{pbo}}\cdot time\cdot \exp(\eta )}\right)$$ (4)

Drug effect (*E*_drug_) is a function dependent on dose, time, fixed-effect model parameters, and trial covariates X. The potential time-varying drug effect was described by an exponential model and the dose-response relationship function was described by a sigmoidal E_max_ model (Eq. 5).

$$E_{\text{drug}}=\;\frac{E_{\text{max}}\cdot \left(1-e^{-k\cdot time}\right)\cdot dose^{\gamma }}{dose^{\gamma }+ED_{50}^{\gamma }}$$ (5)

where *E*_max_ represents the maximum response for each drug, the parameter *k* is the rate constant describing onset of drug effect, *ED*_50_ is dose achieving 50% of maximal response. These parameters were estimated for each drug separately. For those drugs with limited dose regimes or without noticeable dose-response, the parameter *ED*_50_ was estimated as an unreasonable value or failure. Therefore, the *ED*_50_ was fixed to 0 which assumes different dose of this drug have same efficacy if a better model fit was achieved. The *ED*_50_ is set to 0 means that the drug effect is depended on time and *E*_max_, without a dose-response relationship. The Hill coefficient (*γ*) was fixed to 1 in the model because there was not sufficient dose–response information available for each drug.

The inter-study variability was added to the structure model parameter by using exponential formula (Eq. 6) if minimization of the model was successful.

$${\text{P}}_{\text{ij}}\;={\text{ P}}_{\text{j}}\;\times {\text{e}}^{{\text{η}}_{\text{ij}}}$$ (6)

where P_j_ and P_ij_ represent the typical value of *j*th population prediction of the corresponding model parameter and *i*th individual’ *j*th parameter. The inter-study variability (*η*_ij_) was assumed to follow a log-normal distribution with a mean of 0 and a variance of *ω* ^2^. The residual error model (*ε*) was added which is normally distributed with a mean of 0 and a variance of *σ* ^2^ (Eq. 7). In addition, weight based on the standard error of fitted values was introduced to the error model (Eq. 8).

$${\text{Obs}}_{\text{ijt}}\;=\;P_{\text{ijt}}\;+\text{ Weight}\times \text{ε}$$ (7)

$$\text{Weight }=\;\sqrt{\frac{P_{\text{ijt}}\cdot \left(1-P_{\text{ijt}}\right)}{N_{\text{ij}}}}$$ (8)

where *P*_ijt_ and Obs_ijt_ represents the predicted and observed probability of 75% or 90% improvement in PASI scores at *t*th time in treatment arm *j* of trial *i*. *N*_ij_ is the sample size in each arm of each trial. This setting ensured that large studies had a small weight on parameter estimating.

According to previous publication (Checchio et al., 2017), body weight was an important covariate. Therefore, body weight was included in the model if a better model fit was achieved, as described in Eq. 9.

$${\text{Covariate}}_{\text{effect}}={\left(\frac{\text{Covariate}}{\text{mean}(\text{covariate})}\right)}^{\text{θ}}$$ (9)

where *θ* is the parameter describe the quantitative relationship between covariate and model parameter. If body weight of a study could not obtain, it was set to the median data of the analysis data set.

### Model Evaluation

After model establishment, the goodness-of-fit plots and precisions of the parameter estimates were used to describe the adequacy of the final PASI75 and PASI90 longitudinal model. A visual predictive check (VPC) was used to assess the predictive ability of the final model. A total of 1,000 simulations of the final longitudinal model were performed. The VPC graphically showed the observations and different percentiles of simulated predictions (2.5th, median, and 97.5th percentiles).

### Simulation

Base on the final longitudinal model, 1,000 simulations were conducted to generate the drug response at different time point. The results at week 12 were visualized as median and the 2.5th and 97.5th percentiles.

### Analysis Software

The model development and simulation were performed by using NONMEM (v. 7.3) with first-order conditional estimation method. The plots were generated in R (v. 3.5) and Rstudio (v. 1.1.453).

## Results

### Characteristics of Included Studies

**FIGURE 1:**
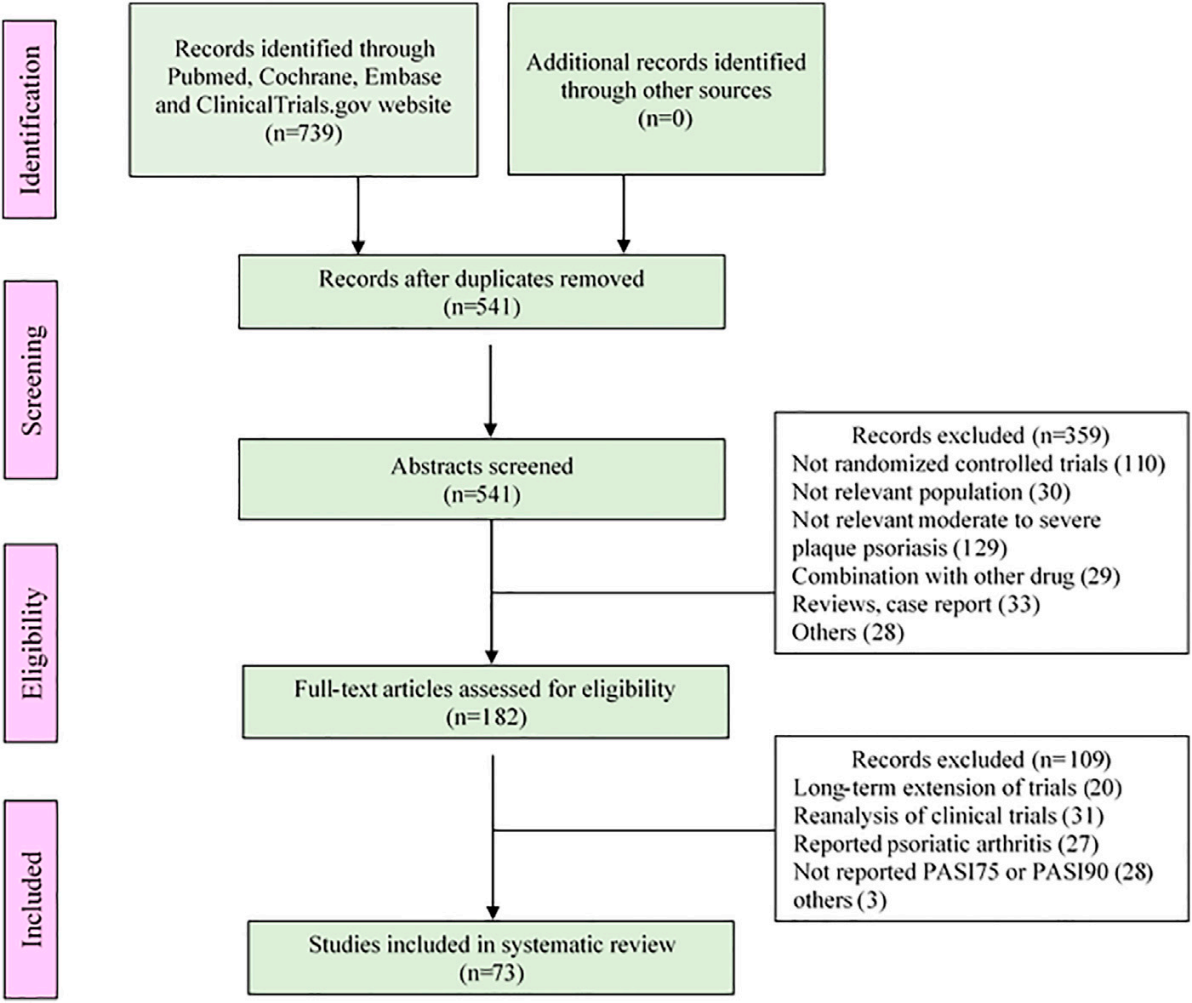
The flow chart of study selection.

The analysis of this study included a total of 80 trials, covering 235 treatment arms and 40,323 patients. The flow chart of the process screening the included studies is presented in Figure 1. All trials were conducted in patients with moderate to severe plaque psoriasis. Among all the treatment arms, PASI75 end point was reported in 233 arms and PASI90 was reported in 224 arms. An outline of the included trials was summarized in Table 1. Detailed information and references are shown in the Supplementary Materials S1.

**TABLE 1:** Summary of available information for each drug in the analysis.

Drug	Trials	Patients	Route (regimen)	Percentage of male (%)^a^	Weight (kg)^a^	Age (year)^a^	Disease duration (years)^a^	Body surface area involved (%)^a^	Baseline PASI^a^	Total Arms	Arms with PASI75	Arms with PASI90
**TNF-α inhibitor**												
Adalimumab	9	2,407	s.c. (40 mg, 80 mg q2w)	70.5 (64.8, 84.2)	86.55 (67.4, 99)	44.05 (42.9, 50)	17.6 (11.6, 21)	29.05 (25, 48.3)	20.95 (14.5, 30.24)	12	12	12
Infliximab	7	1923	i.v. (3 mg/kg 0, 2, 6) i.v. (5 mg/kg 0, 2, 6, q8w)	69 (62.9, 73.7)	84.5 (68.2, 92.2)	44.1 (39.4, 46.9)	18.1 (14.2, 21.5)	28.85 (25, 45.6)	20.4 (17.8, 31.9)	9	9	9
Etanercept	21	4,556	s.c. (25 mg, 50 mg biw)	66 (54, 76.2)	88.35 (74.1, 96.9)	45 (38.6, 55.3)	18.5 (15.2, 23.5)	26.5 (21.3, 33.6)	18.45 (11, 23.2)	25	23	21
Certolizumab pegol	4	810	s.c. (400 mg 0, 2, 4 200 mg q2w) s.c. (400 mg q2w)	68.35 (49.4, 75)	90.75 (83.1, 97.8)	44.95 (43.3, 46.7)	18.7 (16.6, 21)	26.05 (21.4, 28.4)	20.45 (18.4, 22)	8	8	8
**IL-12/23 inhibitor**												
Ustekinumab	11	3,212	s.c. (45 mg, 90 mg 0, 4, q12w)	68 (63.6, 82.8)	90.3 (69.9, 93.8)	45 (40.1, 48.6)	18.7 (11.9, 20.3)	27.1 (20.9, 47)	20.1 (18.2, 30.1)	15	15	15
Briakinumab^c^	6	1,660	s.c. (200 mg 0, 4 100 mg q4w)	71.05 (61, 81)	93.1 (85.1, 96.1)	44.95 (43, 46)	18.3 (16.1, 24)	25.5 (23, 29)	19.05 (18.4, 20)	10	10	10
**IL-23 inhibitor**												
Guselkumab	4	1,161	s.c. (100 mg 0, 4, q8w)	71.4 (67.7, 76.2)	88.7 (67.76, 93.8)	44.5 (41.5, 50.1)	17.9 (14.39, 19.5)	26.2 (21.9, 38)	21.9 (19.4, 26.73)	9	9	9
Tildrakizumab	3	1,545	s.c. (100 mg, 200 mg 0, 4, q12w)	72.5 (65, 85)	88.7 (88.35, 89.35)	45.05 (43.2, 46.9)	NA	31.35 (29.7, 34.2)	20.25 (19.8, 20.7)	8	8	8
Risankizumab	6	1,502	s.c. (150 mg 0, 4, q12w)	70 (69, 91)	87.8 (73, 92.2)	48.65 (45, 53.3)	NA	26.3 (21.8, 41.6)	20.5 (19, 26.9)	8	8	8
**IL-17 inhibitor**												
Secukinumab	5	1710	s.c. (150 mg, 300 mg 0, 1, 2, 3, 4, q4w)	68.5 (64.4, 76.7)	88.8 (83, 93.7)	45.1 (43.9, 46.6)	18 (15.8, 21)	32.8 (26.4, 34.5)	22 (18.9, 23.9)	9	9	9
Ixekizumab	5	2,585	s.c. (80 mg, 160 mg q4w)	66 (50, 70)	92 (85.8, 97)	46 (42.7, 48)	18 (13, 21)	26.7 (21, 28)	19.9 (17.2, 21)	11	11	11
Brodalumab	5	3,189	s.c. (140 mg, 210 mg 0, 1, 2, q2w)	71 (56, 87.2)	90.4 (72.59, 92.4)	45 (42.1, 46.4)	19 (13.32, 20.7)	27 (21.3, 43.7)	20.3 (17.9, 28.53)	13	13	13
**PDE4 inhibitor**												
Apremilast	6	1,525	p.o. (10 mg, 20 mg, 30 mg b.i.d.)	65.8 (57, 83.5)	90.65 (70.1, 95.9)	45.9 (44.1, 52.2)	19.15 (12.6, 20.7)	26.3 (20.7, 32)	19 (18.1, 22.1)	10	10	8
**JAK inhibitor**												
Tofacitinib^b^	6	2,557	p.o. (5mg,10 mg b.i.d.)	72 (59.2, 81.8)	85 (66.6, 93.1)	44 (40.7, 50.9)	16 (13.4, 17)	29.8 (24, 43.1)	21.2 (19.3, 26.9)	13	13	13
Baricitinib^b^	1	237	p.o. (2 mg, 4 mg, 8 mg, 10 mg qd)	72.9 (71.9, 75)	91.15 (89.8, 94.5)	47.4 (47.2, 47.8)	16.6 (15, 19.9)	28.4 (24.5, 30.8)	20.4 (19, 21.4)	4	4	4
**CD2 antagonist**												
Alefacept	1	339	i.m. (10 mg, 15 mg qw)	NA	NA	NA	19 (19, 19)	21 (20, 22)	14 (13, 15)	2	2	0
**Dihydrofolate reductase inhibitor**												
Methotrexate	3	488	p.o. (20 mg qw)	68.1 (66.4, 69)	83.1 (82, 83.8)	41.9 (41.6, 43.1)	18.9 (17, 19.1)	31 (26.1, 32.4)	19.4 (17.8, 21.1)	3	3	3
Placebo	66	8,917		69.2 (52, 89.1)	89.25 (67, 96.5)	45 (39.2, 50.9)	17.9 (11.1, 21)	27.7 (19, 50.2)	19.9 (14, 33.1)	66	66	63
Total	80	40,323		69.05 (49.4, 91)	89.6 (66.6, 99)	45 (38.6, 55.3)	18 (11.1, 24)	27.5 (19, 50.2)	20 (11, 33.1)	235	233	224

PASI, Psoriasis Area and Severity Index score; PASI75, ≥75% reduction from baseline Psoriasis Area and Severity Index score; PASI90, ≥90% reduction from baseline Psoriasis Area and Severity Index score; TNF, tumor necrosis factor; IL, interleukin; PDE, phosphodiesterase; JAK, Janus kinase; NA, not available; s. c., subcutaneous; p. o., oral; qw, once weekly; q2w, once every 2 weeks; q4w, once every 4 weeks; q8w, once every 8 weeks; q12w, once every 12 weeks; biw, twice weekly; qd, once daily.

a Data are shown as median (range).

b Investigational.

c Discontinued. All other drugs are approved.

The drugs included in the analysis contained thirteen biological agents and four small targeted molecules. According to the types of drug targets, it can be divided into the following categories: TNF-α inhibitors (adalimumab, infliximab, etanercept and certolizumab pegol), IL-12/23 inhibitors (ustekinumab and briakinumab), IL-23 inhibitors (guselkumab, tildrakizumab and risankizumab), IL-17 inhibitors (secukinumab, ixekizumab and brodalumab), phosphodiesterase 4 inhibitor (apremilast), Janus kinase inhibitors (tofacitinib and baricitinib), CD2 antagonist (alefacept) and dihydrofolate reductase inhibitor (methotrexate). All drugs were incorporated in the PASI75 longitudinal model. However, alefacept was not included in the PASI90 longitudinal model because of insufficient data.

### PASI75 Model and Typical Drug Efficacies

The PASI75 longitudinal model established in this study could well describe the time-varying drug effect and dose-response relationship. The parameter estimates of drugs for the PASI75 model are provided in Table 2. In the structural model, *E*_drug_ is an exponential function dependent on time. The parameter *E*_max_ represents the maximum efficacy, and the parameter *k* is the rate constant describing the onset of each drug. The *E*_max_ for apremilast was fixed, otherwise the estimation for apremilast showed larger RSE%. For each of the drug, the parameter *k* was estimated with an acceptable estimation accuracy. *ED*_50_ (dose achieving 50% of maximal effect) showed the potency of each drug. For risankizumab, alefacept and methotrexate, the dose-response relationships were not obvious. Therefore, the *ED*_50_ for these drugs was fixed to 0. For all the drugs except apremilast, the dose regimen is higher than *ED*_50_. Take adalimumab for example, the *ED*_50_ value was estimated to be 23.1 mg and the clinical dosage is 40 mg every 2 weeks which means the drug effect is easy to access maximum effect. The placebo effect for PASI75 model was also estimated which was shown in Supplementary Table S1. Body weight effect was included in the parameter *A* (Asymptote of placebo effect) in the PASI75 placebo effect model which resulted in a better model fit.

**TABLE 2:** Final parameter estimates of PASI75 longitudinal model.

Drug	*E*_max_		*ED*_50_		*k*	
	Estimate (RSE%)	95% CI	Estimate (RSE%)	95% CI	Estimate (RSE%)	95% CI
Adalimumab	5.84 (3.7)	(5.417, 6.263)	23.1 (10)	(18.572, 27.628)	0.463 (14.1)	(0.335, 0.591)
Infliximab	4.46 (7)	(3.845, 5.075)	0.462 (48.9)	(0.019, 0.905)	0.663 (13)	(0.494, 0.832)
Etanercept	4.34 (10)	(3.485, 5.195)	21.5 (30.2)	(8.76, 34.24)	0.282 (10.7)	(0.223, 0.341)
Certolizumab pegol	3.95 (3.4)	(3.689, 4.211)	10.1 (70.2)	(−3.796, 23.996)	0.327 (21.7)	(0.188, 0.466)
Ustekinumab	4.33 (4.4)	(3.956, 4.704)	6.38 (41.5)	(1.186, 11.574)	0.241 (10.5)	(0.192, 0.29)
Briakinumab	4.94 (5)	(4.458, 5.422)	12.1 (27)	(5.691, 18.509)	0.317 (8.4)	(0.265, 0.369)
Guselkumab	4.63 (2.4)	(4.416, 4.844)	2.75 (4)	(2.536, 2.964)	0.294 (11.1)	(0.23, 0.358)
Tildrakizumab	3.81 (4)	(3.51, 4.11)	4.63 (9.5)	(3.772, 5.488)	0.229 (10.5)	(0.182, 0.276)
Risankizumab	5.05 (1.8)	(4.872, 5.228)	0 FIX	—	0.242 (9.5)	(0.197, 0.287)
Secukinumab	5.74 (5.4)	(5.136, 6.344)	69.2 (10.4)	(55.108, 83.292)	0.509 (8)	(0.429, 0.589)
Ixekizumab	5.05 (2.3)	(4.821, 5.279)	9.74 (12)	(7.447, 12.033)	1.16 (9.2)	(0.95, 1.37)
Brodalumab	7.69 (8.4)	(6.43, 8.95)	152 (16.6)	(102.412, 201.588)	1.14 (7.5)	(0.973, 1.307)
Apremilast	9 FIX	—	98.5 (6.1)	(86.662, 110.338)	0.381 (20.8)	(0.226, 0.536)
Tofacitinib	4.65 (6.2)	(4.089, 5.211)	4.23 (13.4)	(3.121, 5.339)	0.498 (13.1)	(0.37, 0.626)
Baricitinib	5.63 (19.2)	(3.513, 7.747)	8.4 (20.4)	(5.048, 11.752)	0.24 (42.1)	(0.042, 0.438)
Alefacept	1.38 (5.5)	(1.231, 1.529)	0 FIX	—	0.115 (6.8)	(0.1, 0.13)
Methotrexate	2.32 (4.1)	(2.133, 2.507)	0 FIX	—	0.212 (28.9)	(0.092, 0.332)

**FIGURE 2:**
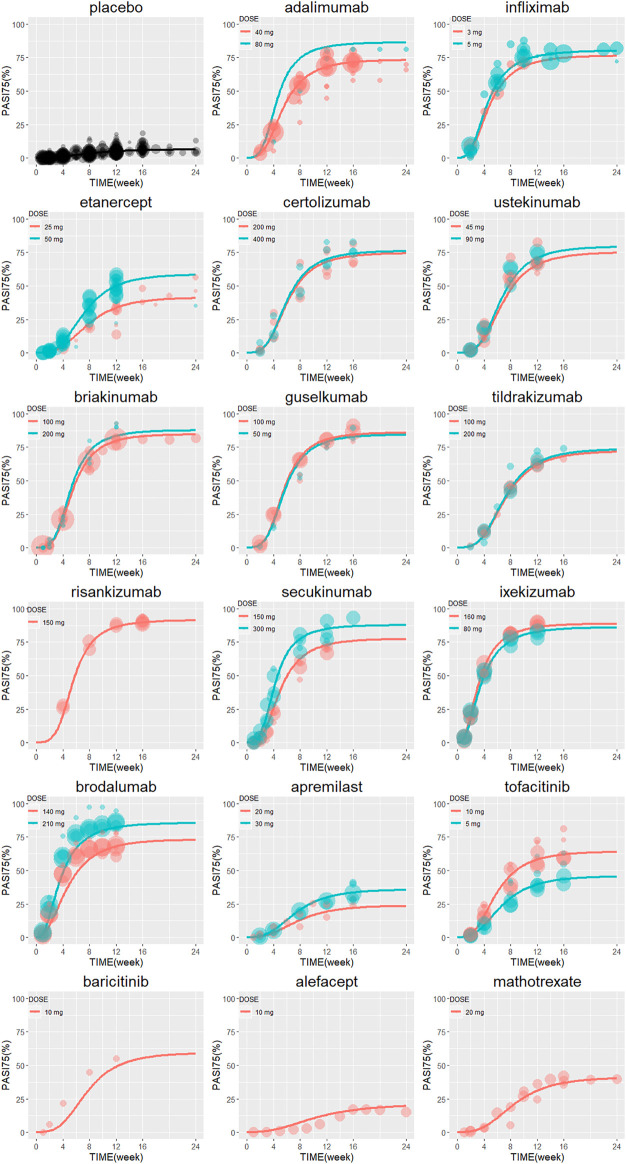
The model predicted typical time course of PASI75 response of each drug. Lines are model predictions for average body weight 90 kg subjects. Circles represent the observed efficacy data, and the symbol size is proportional to the sample size. PASI75, ≥75% reduction from baseline Psoriasis Area and Severity Index score.

**FIGURE 3:**
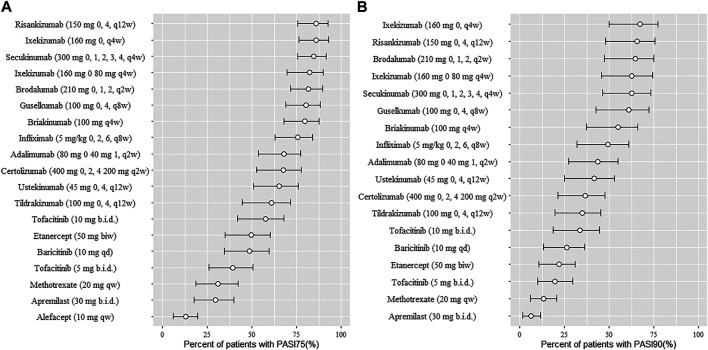
Ranking of treatments for psoriasis presented as median response rate for PASI75 **(A)** and PASI90 **(B)** at week 12 (from high to low). Circles represent the median value and horizontal bars are 95% intervals from model simulation (*N* = 1,000) assuming average body weight 90 kg; PASI75, ≥75% reduction from baseline Psoriasis Area and Severity Index score; PASI90, ≥90% reduction from baseline Psoriasis Area and Severity Index score; qw, once weekly; q2w, once every 2 weeks; q4w, once every 4 weeks; q8w, once every 8 weeks; q12w, once every 12 weeks; biw, twice weekly; qd, once daily; b. i.d., twice daily.

The typical drug efficacies deserved more attention for clinicians. Based on the final PASI75 longitudinal model, the simulated typical PASI75 response-time curves under clinical dose were shown in Figure 2. For each drug, the predicted response-time relationship could cover most of the observed data. As can be seen from the curves, the PASI75 response increased over time and finally reached to the platform. A significant response-dose correlation was observed for adalimumab, etanercept, secukinumab, brodalumab, apremilast and tofacitinib. The drug responses at week 4, 8, 12, 16 and 24 were predicted by 1,000 simulations assuming a body weight value of 90. Results were presented as median PASI75 value together with their 95% intervals in Table 3. Among the IL-23 inhibitors, the PASI75 at week 24 for guselkumab, tildrakizumab and risankizumab were 85.8% (95% confidence interval (CI), 79.4–92.2%), 71.2% (95%CI, 62.6–79.4%) and 91% (95%CI, 85.9–96%), respectively. Risankizumab showed the best response at week 24 not only in IL-23 inhibitors but also in all the treatment. In clinical trials of psoriasis, week 12 is usually a major clinical endpoint. The comparison of all the drugs at week 12 were conducted and summarized in Figure 3A. Of all the therapeutic drugs, risankizumab 150 mg 0, 4, q12w showed the most efficacious with PASI75 was 85.95% (95%CI, 75.71–92.60%), followed by ixekizumab 160 mg 0, q4w with PASI75 was 85.9% (95%CI, 76.12–92.79%). The PASI75 values for secukinumab 300 mg 0, 1, 2, 3, 4, q4w, ixekizumab 160 mg 0, 80 mg q4w, brodalumab 210 mg 0, 1, 2, q2w and guselkumab 100 mg 0, 4, q8w were 84.50% (95%CI, 75.7–91.7%), 82.4% (95%CI, 69.5–89.8%), 81.7% (95%CI, 71.7–89.5%) and 80.4% (95%CI, 69–88.3%), respectively, which ranked from third to sixth. Overall, the IL-17 inhibitors and IL-23 inhibitors except tildrakizumab were more effective than other classes of biologics and small molecules. Among the TNF-α inhibitors, infliximab 5 mg/kg 0, 2, 6, q8w showed the best response with PASI75 was 75.65% (95%CI, 62.8–84%). In small molecule drugs, a significant difference was only found in tofacitinib 10 mg b.i.d. with PASI75 was 57.7% (95%CI, 41.9–68%). Alefacept showed less efficacious than other treatments.

**TABLE 3:** Model predicted PASI75 response of treatments at different time points.

Drug	Regimen	PASI75(%)^a^				
		Week 4	Week 8	Week 12	Week 16	Week 24
TNF-α inhibitor						
Adalimumab	80 mg 0 40 mg 1, q2w	21.30 (8.59, 39.20)	56.95 (36.30, 70.40)	67.80 (53.70, 77.30)	71.20 (61.31, 79.90)	72.80 (65.20, 80.10)
Infliximab	5 mg/kg 0, 2, 6, q8w	34.10 (16.60, 55.20)	67.10 (47.70, 80.20)	75.65 (62.80, 84.00)	78.20 (69.40, 85.40)	79.70 (72.71, 86.40)
Etanercept	50 mg biw	7.98 (2.29, 17.90)	34.40 (18.70, 48.80)	49.60 (34.91, 60.20)	55.00 (43.50, 64.40)	57.80 (48.30, 66.70)
Certolizumab pegol	400 mg 0, 2, 4 200 mg q2w	15.30 (5.30, 31.20)	53.15 (33.72, 68.60)	67.70 (52.61, 77.69)	71.80 (62.01, 80.40)	74.00 (67.00, 81.30)
IL-12/23 inhibitor						
Ustekinumab	45 mg 0, 4, q12w	10.75 (3.36, 23.70)	47.30 (28.10, 61.90)	65.30 (50.90, 75.80)	71.20 (61.30, 80.70)	74.50 (66.60, 81.90)
Briakinumab	100 mg q4w	21.50 (8.78, 41.70)	66.80 (46.91, 80.20)	79.50 (67.81, 87.70)	82.80 (74.60, 90.10)	84.40 (77.80, 91.10)
IL-23 inhibitor						
Guselkumab	100 mg 0, 4, q8w	20.65 (8.40, 38.19)	67.00 (47.21, 79.70)	80.40 (69.00, 88.30)	83.90 (76.30, 90.40)	85.80 (79.40, 92.20)
Tildrakizumab	100 mg 0, 4, q12w	9.26 (2.33, 21.40)	42.80 (22.70, 57.70)	61.00 (44.71, 71.60)	68.10 (56.90, 76.20)	71.20 (62.60, 79.40)
Risankizumab	150 mg 0, 4, q12w	21.15 (8.78, 40.10)	72.50 (53.31, 83.90)	85.95 (75.71, 92.60)	89.40 (82.90, 94.70)	91.00 (85.90, 96.00)
IL-17 inhibitor						
Secukinumab	300 mg 0, 1, 2, 3, 4, q4w	39.60 (20.00, 62.60)	77.20 (58.90, 87.50)	84.50 (75.70.91.70)	86.70 (79.40, 93.10)	87.60 (82.20, 93.60)
Ixekizumab	160 mg 0, q4w	56.50 (33.50, 76.60)	80.40 (62.80, 89.40)	85.90 (76.12, 92.79)	87.70 (80.50, 93.10)	88.50 (82.50, 93.90)
Ixekizumab	160 mg 0 80 mg q4w	48.95 (26.90, 70.39)	75.20 (56.50, 85.80)	82.40 (69.50, 89.80)	84.60 (77.10, 90.90)	85.70 (79.90, 91.50)
Brodalumab	210 mg 0, 1, 2, q2w	48.55 (27.20, 70.50)	74.95 (56.60, 85.50)	81.70 (71.70, 89.50)	84.00 (76.30, 90.10)	85.20 (79.10, 91.30)
PDE4 inhibitor						
Apremilast	30 mg b.i.d.	5.68 (0.92, 13.40)	20.30 (9.18, 32.69)	29.50 (17.50, 39.79)	33.20 (23.30, 41.70)	35.30 (27.31, 43.50)
JAK inhibitor						
Tofacitinib	5 mg b.i.d.	9.07 (2.48, 19.90)	29.00 (14.40, 42.40)	39.20 (25.90, 50.50)	42.80 (32.30, 51.60)	45.10 (36.30, 53.90)
Tofacitinib	10 mg b.i.d.	16.30 (5.52, 32.50)	46.55 (26.50, 62.00)	57.70 (41.90, 68.00)	61.60 (51.00, 70.20)	63.10 (54.41, 72.30)
Baricitinib	10 mg qd	7.34 (1.66, 16.60)	33.10 (17.10, 46.30)	48.70 (34.40, 59.50)	54.80 (44.10, 64.10)	58.30 (49.91, 67.10)
CD2 antagonist						
Alefacept	10 mg qw	1.90 (0, 5.85)	7.46 (1.83, 14.50)	12.90 (5.90, 19.60)	15.90 (9.40, 23.20)	19.30 (12.60, 26.20)
Dihydrofolate reductase inhibitor						
Methotrexate	20 mg qw	4.14 (0.48, 10.50)	19.00 (7.61, 30.30)	30.80 (18.51, 42.10)	36.80 (25.80, 46.30)	40.10 (31.80, 48.30)

PASI, Psoriasis Area and Severity Index score; PASI75, ≥75% reduction from baseline Psoriasis Area and Severity Index score; TNF, tumor necrosis factor; IL, interleukin; PDE, phosphodiesterase; JAK, Janus kinase; NA, not available; s.c., subcutaneous; p. o., oral; qw, once weekly; q2w, once every 2 weeks; q4w, once every 4 weeks; q8w, once every 8 weeks; q12w, once every 12 weeks; biw, twice weekly; qd, once daily.

a Data are shown as median (95% CI).

### PASI90 Model and Typical Drug Efficacies

Similar to the PASI75 model, the time-varying drug effect and dose-effect relationship in PASI90 endpoint were well described by an exponential model and E_max_ model, respectively. The final estimated parameters of PASI90 longitudinal model are listed in Table 4. The dose-response difference of ustekinumab was not significant and estimating *ED*_50_ resulted in a poor model estimation accuracy. Therefore, *ED*_50_ for ustekinumab was fixed to 0 subsequently. The *ED*_50_ values of guselkumab, tildrakizumab and risankizumab were 2.95 mg (95%CI, 2.578–3.322), 7.16 mg (95%CI, 3.534–10.786) and 13.3 mg (95%CI, 7.067–19.533), respectively. The placebo effect for PASI90 model was estimated and the results were listed in Supplementary Table S2. Body weight showed an effect on placebo component *A* of the PASI90 placebo effect model which was consistent as PASI75 model.

**TABLE 4:** Final parameter estimates of PASI90 longitudinal model.

Drug	*E*_max_		*ED*_50_		*k*	
	Estimate (RSE%)	95% CI	Estimate (RSE%)	95% CI	Estimate (RSE%)	95% CI
Adalimumab	5.74 (7.6)	(4.885, 6.595)	18.3 (25.7)	(9.088, 27.512)	0.487 (26.5)	(0.234, 0.74)
Infliximab	4.73 (4.6)	(4.307, 5.153)	0.689 (23.8)	(0.368, 1.01)	0.802 (35.2)	(0.249, 1.355)
Etanercept	5.33 (11.2)	(4.158, 6.502)	30.7 (25.3)	(15.471, 45.929)	0.181 (12.7)	(0.136, 0.226)
Certolizumab pegol	3.99 (4.9)	(3.608, 4.372)	8.1 (63.2)	(−1.935, 18.135)	0.242 (21.2)	(0.141, 0.343)
Ustekinumab	4.06 (3.3)	(3.795, 4.325)	0 FIX	—	0.243 (11.6)	(0.188, 0.298)
Briakinumab	4.9 (4)	(4.518, 5.282)	9.47 (20.1)	(5.746, 13.194)	0.341 (14.3)	(0.245, 0.437)
Guselkumab	4.78 (3.8)	(4.427, 5.133)	2.95 (6.4)	(2.578, 3.322)	0.537 (43)	(0.084, 0.99)
Tildrakizumab	3.99 (5.4)	(3.571, 4.409)	7.16 (25.8)	(3.534, 10.786)	0.252 (45.6)	(0.027, 0.477)
Risankizumab	5.56 (2.8)	(5.252, 5.868)	13.3 (23.9)	(7.067, 19.533)	0.246 (9.4)	(0.201, 0.291)
Secukinumab	5.98 (5.1)	(5.382, 6.578)	79.2 (15.4)	(55.288, 103.112)	0.467 (16.1)	(0.32, 0.614)
Ixekizumab	5.14 (3.3)	(4.809, 5.471)	7.02 (15.8)	(4.844, 9.196)	1.12 (22.5)	(0.626, 1.614)
Brodalumab	6.92 (7.7)	(5.875, 7.965)	92.5 (20.4)	(55.456, 129.544)	1.19 (16)	(0.818, 1.562)
Apremilast	8.8 FIX	—	54.1 (52.3)	(−1.368, 109.568)	0.0541 (50.1)	(0.001, 0.107)
Tofacitinib	4.76 (10.7)	(3.758, 5.762)	3.42 (31.9)	(1.284, 5.556)	0.395 (62.8)	(−0.091, 0.881)
Baricitinib	4.1 (4.4)	(3.749, 4.451)	2.9 (14.1)	(2.096, 3.704)	0.481 (24.7)	(0.248, 0.714)
Methotrexate	2.56 (5.6)	(2.28, 2.84)	0 FIX	—	0.19 (32.6)	(0.068, 0.312)

**FIGURE 4:**
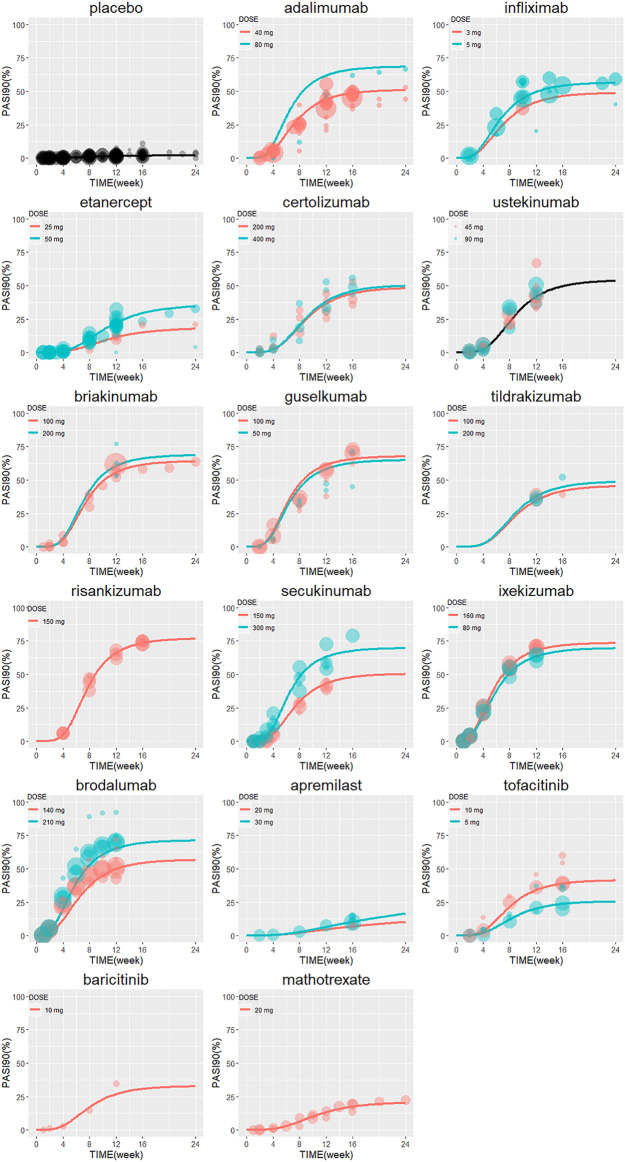
The model predicted typical time course of PASI90 response of each drug. Lines are model predictions for average body weight 90 kg subjects. Circles represent the observed efficacy data, and the symbol size is proportional to the sample size. PASI90, ≥90% reduction from baseline Psoriasis Area and Severity Index score.

The final PASI90 model was used to simulate the percentage of person achieve PASI90 at different time point, assuming a typical body weight with 90 kg. The typical response-time course and dose–effect correlation of PASI90 end point after different treatment was shown in Figure 4. The trend of the PASI90 response-time curve is approximate as PASI75 curve. The response-dose correlations of adalimumab, infliximab, etanercept, secukinumab, brodalumab, and tofacitinib were significant. The simulation results of PASI90 response at week 4, 8, 12, 16 and 24 were summarized in Table 5. For the IL-23 inhibitors, risankizumab showed the best response at week 24 with a value of 76.7% (95%CI, 69.7–84%), followed by guselkumab with PASI90 response 67.4% (95%CI, 58.7–75.5%). The tildrakizumab showed the least PASI90 response with a value of 44.9% (95%CI, 36.7–53.9%). Comparation of PASI90 response at week 12 were presented in Figure 3B. It reveals that risankizumab, ixekizumab, secukinumab, brodalumab and guselkumab were still the most effective treatment, which was similar to the results of PASI75 model. Ixekizumab 160 mg 0, q4w showed the greatest percentage of person achieved PASI90 (67.2%; 95%CI, 49.91–77.2%) at week 12, followed by risankizumab 150 mg 0, 4, q12w (65.5%; 95%CI, 47.8–75.7%). The percentage of person achieved PASI90 for brodalumab 210 mg 0, 1, 2, q2w, ixekizumab 160 mg 0, 80 mg q4w, secukinumab 300 mg 0, 1, 2, 3, 4, q4w and guselkumab 100 mg 0, 4, q8w were 64.5% (95%CI, 47.31–75%), 62.6% (95%CI, 45.4–74.2%), 62.5% (95%CI, 46.1–73.3%) and 61% (95%CI, 42.5–72.1%), respectively. For the PASI90 end point, the IL-17 inhibitors and IL-23 inhibitors except tildrakizumab were also more effective than other classes of biologics and small molecules. On the whole, the responses of biological agents except etanercept were super than small molecules which was reflected in the figure. Tofacitinib 10 mg b.i.d. (33.6%; 95%CI, 18.7–44.5%) was the most effective in small molecule drugs. Apremilast showed less efficacious than other treatments.

**TABLE 5:** Model predicted PASI90 response of treatments at different time points.

Drug	Regimen	PASI90(%)^a^				
		Week 4	Week 8	Week 12	Week 16	Week 24
TNF-α inhibitor						
Adalimumab	80 mg 0 40 mg 1, q2w	6.10 (0.69, 18.00)	30.30 (12.10, 48.59)	43.50 (27.11, 55.00)	48.05 (35.70, 58.20)	50.60 (42.70, 59.90)
Infliximab	5 mg/kg 0, 2, 6, q8w	10.40 (2.45, 26.80)	36.80 (14.81, 54.20)	49.15 (31.81, 61.00)	53.40 (40.60, 63.80)	55.90 (46.80, 65.00)
Etanercept	50 mg biw	1.16 (0, 4.73)	9.90 (2.90, 20.10)	22.00 (10.60, 31.00)	28.95 (18.20, 37.89)	34.40 (25.90, 42.50)
Certolizumab pegol	400 mg 0, 2, 4 200 mg q2w	2.29 (0, 7.70)	20.00 (6.95, 34.40)	36.50 (21.10, 47.70)	43.50 (31.51, 53.40)	47.80 (39.20, 55.80)
IL-12/23 inhibitor						
Ustekinumab	45 mg 0, 4, q12w	2.64 (0, 8.55)	22.90 (8.72, 38.10)	41.60 (24.91, 52.80)	48.85 (37.20, 58.10)	53.30 (45.00, 62.10)
Briakinumab	100 mg q4w	5.83 (0.71, 16.50)	37.10 (16.41, 54.90)	54.85 (37.11, 65.90)	60.80 (48.81, 70.20)	63.60 (54.40, 71.40)
IL-23 inhibitor						
Guselkumab	100 mg 0, 4, q8w	11.7 (3.08, 30.09)	47.45 (23.60, 65.49)	61.00 (42.50, 72.10)	65.40 (52.30, 74.50)	67.40 (58.70, 75.50)
Tildrakizumab	100 mg 0, 4, q12w	2.21 (0, 6.88)	18.10 (7.01, 31.10)	34.80 (19.40, 45.40)	40.70 (29.90, 50.50)	44.90 (36.70, 53.90)
Risankizumab	150 mg 0, 4, q12w	5.11 (0.41, 15.60)	42.15 (19.60, 60.79)	65.50 (47.80, 75.70)	72.70 (61.41, 81.40)	76.70 (69.70, 84.00)
IL-17 inhibitor						
Secukinumab	300 mg 0, 1, 2, 3, 4, q4w	10.60 (2.04, 26.30)	47.95 (23.92, 65.90)	62.50 (46.10, 73.30)	66.70 (56.10, 75.80)	69.30 (61.40, 77.50)
Ixekizumab	160 mg 0, q4w	21.60 (6.91, 47.79)	55.35 (30.00, 72.70)	67.20 (49.91, 77.20)	71.10 (60.10, 79.50)	73.10 (65.00, 80.60)
Ixekizumab	160 mg 0 80 mg q4w	18.40 (5.91, 45.10)	50.40 (24.01, 67.80)	62.60 (45.40, 74.20)	66.95 (55.20, 76.00)	69.40 (60.71, 77.00)
Brodalumab	210 mg 0, 1, 2, q2w	19.90 (5.81, 44.50)	52.15 (27.00, 70.49)	64.50 (47.31, 75.00)	68.60 (57.30, 78.20)	70.80 (62.80, 79.00)
PDE4 inhibitor						
Apremilast	30 mg b.i.d.	0.38 (0, 2.14)	2.68 (0, 6.36)	6.09 (1.45, 11.50)	9.75 (4.23, 15.30)	16.40 (9.99, 22.60)
JAK inhibitor						
Tofacitinib	5 mg b.i.d.	1.99 (0, 6.89)	12.00 (3.29, 22.60)	19.50 (9.94, 29.40)	23.40 (14.00, 31.70)	25.10 (17.60, 32.50)
Tofacitinib	10 mg b.i.d.	3.52 (0.03, 10.60)	21.40 (7.96, 36.40)	33.60 (18.70, 44.50)	38.15 (26.80, 47.10)	40.70 (31.60, 49.10)
Baricitinib	10 mg qd	3.05 (0.08, 10.50)	17.20 (5.53, 30.10)	26.20 (13.30, 36.20)	29.95 (18.90, 38.70)	32.30 (23.90, 40.60)
Dihydrofolate reductase inhibitor						
Methotrexate	20 mg qw	0.85 (0, 3.59)	6.27 (1.19, 13.80)	13.05 (5.76, 20.70)	17.00 (9.42, 24.30)	20.10 (12.50, 27.10)

PASI, Psoriasis Area and Severity Index score; PASI90, ≥90% reduction from baseline Psoriasis Area and Severity Index score; TNF, tumor necrosis factor; IL, interleukin; PDE, phosphodiesterase; JAK, Janus kinase; NA, not available; s.c., subcutaneous; p.o., oral; qw, once weekly; q2w, once every 2 weeks; q4w, once every 4 weeks; q8w, once every 8 weeks; q12w, once every 12 weeks; biw, twice weekly; qd, once daily.

a Data are shown as median (95% CI).

### Model Evaluation

The goodness-of-fit plots for the PASI75 and PASI90 model are presented in Supplementary Figures S1–S2, which shows there are not any apparent systematic bias and misspecification. The VPC results for the PASI75 and PASI90 model are shown in Supplementary Figures S3–S4. This VPC plots indicated an adequate predictive ability of the final model.

## Discussion

Our study presented a quantitative information for the efficacy comparation across seventeen drugs. Two independent longitudinal model-based meta-analyses including two binary end points (PASI75 and PASI90) were carried out to describe the time-varying drug efficacy. PASI75 end point has previously been benchmark to define treatment response in plaque psoriasis clinical trials (Carlin et al., 2004). However, higher standards (complete or near complete clearance of psoriasis) is now a reality with newer biologic therapies. PASI90 or PASI100 were adopted as the new standard for an optimal response in clinical trials (Puig, 2015). In our study, both PASI75 and PASI90 were included which offered a comprehensive understanding of the drug efficacy. The data of PASI100 was not adequate to build a longitudinal model. Clinicians and patients could select their preferred treatments depending on their priorities.

In general, the efficacy trend is similar across these two endpoints: anti-IL-23 and anti-IL-17 treatments except tildrakizumab were the most effective among the classes of biologics and small molecules. Risankizumab administered as 150 mg at week 0, week 4 and q12w and ixekizumab administered as 160 mg at week 0 followed by 160 mg q4w or 80 mg q2w provided the highest response of PASI75 and PSAI90 at week 12, respectively. Risankizumab administered as 150 mg at week 0, week 4 and q12w also provided the highest response of PASI90 at week 16 and week 24. The efficacy of risankizumab is superior than adalimumab is observed in our study which is also supported by a head-to-head trial and a meta-analysis (Reich et al., 2019; Witjes et al., 2020). Superior efficacy of guselkumab, another IL-23 inhibitor, was also predicted compared with adalimumab which is in concert with previous trials (Blauvelt et al., 2017; Reich, et al., 2017). Although our model-based meta-analysis showed a quantitative comparison of risankizumab and IL-17 inhibitors. However, the efficacy of risankizumab compared with IL-17 inhibitors need to demonstrated by head-to-head trials. A direct comparation of risankizumab and secukinumab is ongoing in a phase 3 study (NCT03478787). The targeted therapies for the treatment of moderate-to-severe plaque psoriasis is going through a re-evaluation.

Compared with the conclusions of previous meta-analyses, our study provided different and more detailed rank-order. The comparison about existing meta-analyses including IL-23 inhibitors are summarized in Supplementary Table S3. Apart from common biologicals of IL-17, IL-23 and TNF-α inhibitors (ustekinumab, secukinumab, brodalumab, ixekizumab, guselkumab, tildrakizumab, adalimumab, infliximab, etanercept, certolizumab pegol) which were generally reported in these meta-analyses, another four small targeted molecules (apremilast, tofacitinib, baricitinib amd methotrexate) were evaluated in our model-based meta-analysis. This is a comprehensive quantitative comparison of systemic medications for moderate to severe plaque psoriasis. Furthermore, the efficacy data at different time were all used by an exponential model to describe the potential time-varying drug effect, whereas the traditional pairwise and network meta-analyses pooled the efficacy data at different time. Our study provided a new insight in the onset of drug response by applying longitudinal data. Next, the dose-response relationships were estimated separately. Finally, in the process of comparing the efficacy of systemic agents, the results of week 4, 8, 12, 16, 24 were conducted and detailed rank-order at week 12 was visualized clearly.

This study has some advantages. First, our analysis was based on longitudinal model. We provided time-course response of all the agents for the treatment of psoriasis. It has the benefits of model-based meta-analysis such as extracting knowledge from all the time point data and different doses, which is quite different from traditional meta-analysis. A difference may be explored (such as superiority of risankizumab) by using the extracted knowledge. Second, our analysis was conducted on the basis of the largest number of medications, trials and patients. Third, trial differences of placebo effect were estimated by adding a between-trials variability parameter (Supplementary Tables S1–S2 **)**, which reduced a common placebo effect parameter across all studies.

There are also some limitations in our analysis. First, we only focused on the efficacy of systemic agents for psoriasis, ignoring the pooled safety profiles. Second, the efficacy data together with time course data in this analysis was not adequate for some treatments which led to some parameters estimation failed such as apremilast, risankizumab (PASI75 model), methotrexate and alefacept or resulted in imprecize estimation such as certolizumab (PASI75 model) and tofacitinib (PASI90 model). In addition, the time of study performance differs a lot. Some previous studies included more severe patients as compared to more recent studies.

In clinical practice, there are more factors to consider. Apart from the efficacy, the cost of drug is another concern. The lower cost biosimilars were also developed. The first issue is that what is the extent of the clinical difference in practice between patent drugs and lower cost biosimilars and how to choose it. Second issue is how to decide from one biologics shift to another. These issues remains unknown and more studies should be conducted in the future. We can provide future direction to patients by getting more evidence.

## Conclusion

In conclusion, the model-based meta-analysis provided quantitative information about the efficacy comparation in psoriasis of five classes of biologics and three classes of small molecules with a total of seventeen systemic agents for two end points in term of efficacy only and that safety was not considered. Risankizumab and ixekizumab showed superiority for both the two end points. Further studies about the long-term efficacy are still needed to enhance the understanding of the relative drug efficacy and safety.

## Data Availability

The raw data supporting the conclusion of this article will be made available by the authors, without undue reservation.
