# Supplementary material for: Model-Based Meta-Analysis in Psoriasis: A Quantitative Comparison of Biologics and Small Targeted Molecules
Source: Front Pharmacol. 2021 Jul 1;12:586827. doi: 10.3389/fphar.2021.586827 (PMC8281289; doi:10.3389/fphar.2021.586827)
Supplement: Supplementary file 1 [file Table1.docx]

Table S1 Parameter estimation of the placebo effect for PASI75 model

| **Parameter** | Estimate (RSE%) | 95% CI |
| --- | --- | --- |
| **Placebo effect** |  |  |
| Intercept of placebo effect (*BSL*) | -7.52 (3.2) | (-7.986, -7.054) |
| Asymptote of placebo effect (*A*) | 4.83 (4.5) | (4.407, 5.253) |
| Rate of onset of placebo effect (*k*_pbo_) | 0.25 (6.4) | (0.218, 0.282) |
| **Covariate** |  |  |
| Body weight effect on *A* | -0.245 (44.1) | (-0.457, -0.033) |
| **Random effect** |  |  |
| ω(*A*),% | 24.9 (21.7) | (14.31, 35.49) |
| σ | 1.33 (7.4) | (1.14, 1.52) |
