# Supplementary material for: Model-Based Meta-Analysis in Psoriasis: A Quantitative Comparison of Biologics and Small Targeted Molecules
Source: Front Pharmacol. 2021 Jul 1;12:586827. doi: 10.3389/fphar.2021.586827 (PMC8281289; doi:10.3389/fphar.2021.586827)
Supplement: Supplementary file 2 [file Image4.PDF]

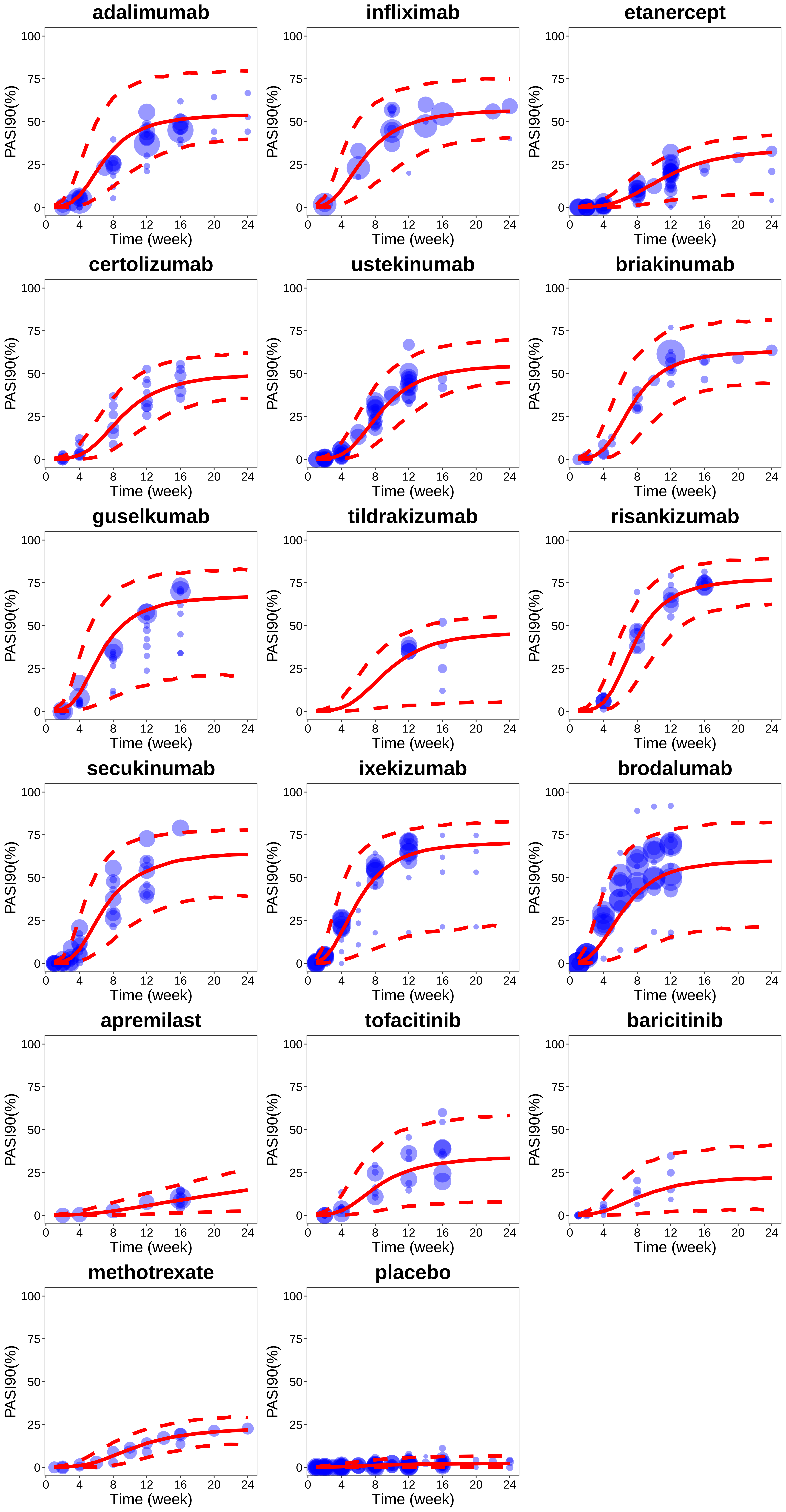

adalimumab
infliximab
etanercept
certolizumab
ustekinumab
briakinumab
guselkumab
tildrakizumab
risankizumab
secukinumab
ixekizumab
brodalumab
apremilast
tofacitinib
baricitinib
methotrexate
placebo
PASI90(%)
Time (week)
0
25
50
75
100
0
4
8
12
16
20
24
