# Supplementary material for: Model-Based Meta-Analysis in Psoriasis: A Quantitative Comparison of Biologics and Small Targeted Molecules
Source: Front Pharmacol. 2021 Jul 1;12:586827. doi: 10.3389/fphar.2021.586827 (PMC8281289; doi:10.3389/fphar.2021.586827)
Supplement: Supplementary file 5 [file Table2.docx]

Table S2 Parameter estimation of the placebo effect for PASI90 model

| **Parameter** | Estimate (RSE%) | 95% CI |
| --- | --- | --- |
| **Placebo effect** |  |  |
| Intercept of placebo effect (*BSL*) | -10.2 (6.3) | (-11.456, -8.944) |
| Asymptote of placebo effect (*A*) | 6.32 (9.5) | (5.148, 7.492) |
| Rate of onset of placebo effect (*k*_pbo_) | 0.259 (7.4) | (0.222, 0.296) |
| **Covariate** |  |  |
| Body weight effect on *A* | -0.214 (33.6) | (-0.355, -0.073) |
| **Random effect** |  |  |
| ω(*A*),% | 26 (24.9) | (13.3, 38.7) |
| σ | 1.33 (9.7) | (1.08, 1.58) |
