# Supplementary material for: Model-Based Meta-Analysis in Psoriasis: A Quantitative Comparison of Biologics and Small Targeted Molecules
Source: Front Pharmacol. 2021 Jul 1;12:586827. doi: 10.3389/fphar.2021.586827 (PMC8281289; doi:10.3389/fphar.2021.586827)
Supplement: Supplementary file 6 [file Table3.docx]

Table S3 Comparison of the previous meta-analyses

| Studies | Included drugs | Article types | Characteristics | Ranking based PASI75  (From high to low) | Ranking based PASI90  (From high to low) |
| --- | --- | --- | --- | --- | --- |
| Erichsen CY, 2020^1^ | Ustekinumab  Ixekizumab  Secukinumab  Brodalumab  Guselkumab  Tildrakizumab  Risankizumab | Traditional pairwise  meta- analysis | Only included end point at week 12 or week 16;  Efficacy was assessed together, not separately; | —— | (Compared against placebo,week12 or 16)  Ixekizumab q2w  Ixekizumab q4w  Secukinumab 300mg  Secukinumab 150mg  Brodalumab 210mg  Ustekinumab 90mg  Guselkumab 100mg  Brodalumab 140mg  Risankizumab 150mg  Tildrakizumab 200mg  Tildrakizumab 100mg |
| Warren RB, 2020^2^ | Secukinumab  Brodalumab  Ixekizumab  Ustekinumab  Guselkumab  Tildrakizumab  Infliximab  Adalimumab  Etanercept | Network  meta- analysis | Pooled efficacy data at week 12 or 16;  Calculations of cumulative clinical benefits; | (week 12)  Ixekizumab (80 mg q2w)  Brodalumab 210 mg  Ixekizumab (80 mg q4w)  Secukinumab 300 mg  Brodalumab 140 mg  Infliximab 5 mg/kg  Guselkumab 100 mg  Secukinumab 150 mg  Ustekinumab 90mg  Adalimumab 40 mg  Ustekinumab 45 mg  Tildtakizumab 200 mg  Tildtakizumab 100 mg  Etanercept 50 mg | (week12)  Brodalumab 210 mg  Ixekizumab (80 mg q2w)  Ixekizumab (80 mg q4w)  Brodalumab 140 mg  Infliximab 5 mg/kg  Secukinumab 300 mg  Guselkumab 100 mg Adalimumab 40 mg  Ustekinumab 90mg  Secukinumab 150 mg  Ustekinumab 45 mg  Tildtakizumab 200 mg  Tildtakizumab 100 mg  Etanercept 50 mg |
| Armstrong AW, 2020^3^ | Ustekinumab  Secukinumab  Brodalumab  Ixekizumab  Guselkumab  Tildrakizumab  Infliximab  Adalimumab  Etanercept  Certolizumab  Risankizumab  Apremilast | Network  meta- analysis | At the end of the primary response period (10-16  weeks from baseline; | (Short-term Efficacy)  Risankizumab-rzaa, 150 mg  Ixekizumab, 80 mg  Brodalumab, 210 mg  Guselkumab, 100 mg  Secukinumab, 300 mg  Infliximab, 5 mg/kg  Certolizumab pegol, 400 mg  Ustekinumab, 45 mg ≤100 kg, 90 mg >100 kg  Adalimumab, 40 mg  Certolizumab pegol, 200 mg  Tildrakizumab-asmn, 200 mg  Tildrakizumab-asmn, 100 mg  Etanercept, 25 mg twice weekly/50 mg once weekly  Apremilast, 30 mg | (Short-term Efficacy)  Risankizumab-rzaa, 150 mg  Ixekizumab, 80 mg  Brodalumab, 210 mg  Guselkumab, 100 mg  Secukinumab, 300 mg  Infliximab, 5 mg/kg  Certolizumab pegol, 400 mg  Ustekinumab, 45 mg ≤100 kg, 90 mg >100 kg  Adalimumab, 40 mg  Certolizumab pegol, 200 mg  Tildrakizumab-asmn, 200 mg  Tildrakizumab-asmn, 100 mg  Etanercept, 25 mg twice weekly/50 mg once weekly  Apremilast, 30 mg |

1 Erichsen CY, Jensen P, Kofoed K. Biologic therapies targeting the interleukin (IL)-23/IL-17 immune axis for the treatment of moderate-to-severe plaque psoriasis: a systematic review and meta-analysis. *J Eur Acad Dermatol Venereol* 2020; **34**: 30-38.

2 Warren RB, Gooderham M, Burge R *et al.* Comparison of cumulative clinical benefits of biologics for the treatment of psoriasis over 16 weeks: Results from a network meta-analysis. *J Am Acad Dermatol* 2020; **82**: 1138-1149.

3 Armstrong AW, Puig L, Joshi A *et al.* Comparison of Biologics and Oral Treatments for Plaque Psoriasis: A Meta-analysis. *JAMA Dermatol* 2020; : .
