# Supplementary material for: Model-Based Meta-Analysis in Psoriasis: A Quantitative Comparison of Biologics and Small Targeted Molecules
Source: Front Pharmacol. 2021 Jul 1;12:586827. doi: 10.3389/fphar.2021.586827 (PMC8281289; doi:10.3389/fphar.2021.586827)
Supplement: Supplementary file 7 [file Image1.PDF]

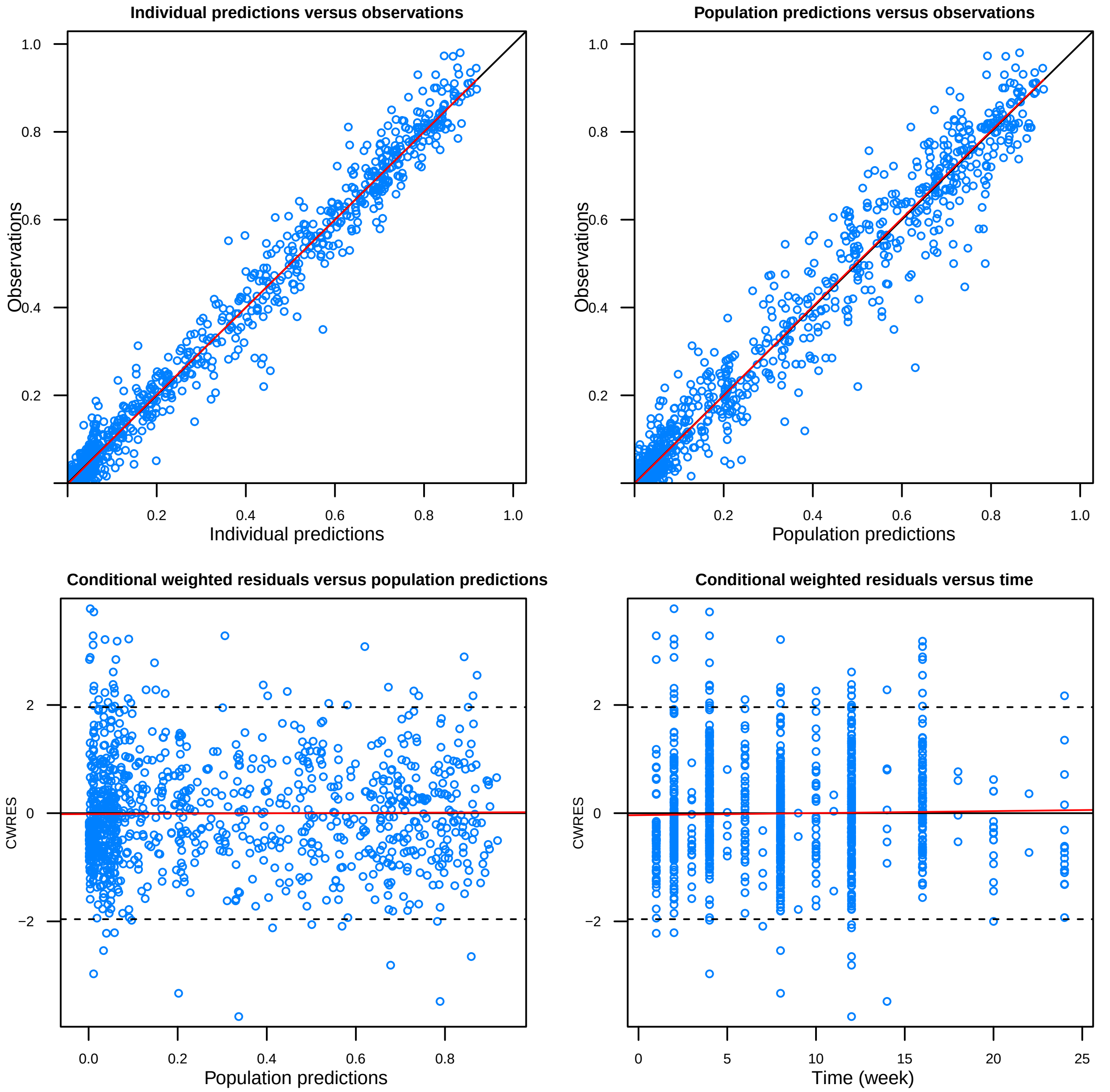

Individual predictions versus observations
Observations
Individual predictions
Population predictions versus observations
Observations
Population predictions
Conditional weighted residuals versus population predictions
CWRES
Population predictions
Conditional weighted residuals versus time
CWRES
Time (week)
